# Look Local: The Value of Cancer Surveillance and Reporting by American Indian Clinics

**DOI:** 10.5888/pcd10.130153

**Published:** 2013-11-27

**Authors:** Paul D. Creswell, Rick Strickland, Laura Stephenson, Kimmine Pierce-Hudson, Jacqueline Matloub, Jerry Waukau, Alexandra Adams, Judith Kaur, Patrick L. Remington

**Affiliations:** Author Affiliations: Paul D. Creswell, University of Wisconsin Department of Population Health Sciences, Madison, Wisconsin; Laura Stephenson, Division of Public Health, Department of Health Services, Madison, Wisconsin; Kimmine Pierce-Hudson, Indian Health Service, Division of Epidemiology and Disease Prevention, Rockville, Maryland; Jacqueline Matloub, Prevention Research Center for Healthy Neighborhoods, Case Western Reserve University, Cleveland, Ohio; Jerry Waukau, Wisconsin Tribal Health Directors Association, Keshena, Wisconsin; Alexandra Adams, Spirit of EAGLES, University of Wisconsin Carbone Cancer Center, Madison, Wisconsin; Judith Kaur, Spirit of EAGLES, Mayo Clinic Comprehensive Cancer Center, Rochester, Minnesota; Patrick L. Remington, University of Wisconsin Carbone Cancer Center, Madison, Wisconsin. At time of study, Ms Pierce-Hudson worked at Great Lakes Inter-Tribal Epidemiology Center, Great Lakes Inter-Tribal Council, Inc, Lac du Flambeau, Wisconsin, and Ms Matloub worked for Spirit of EAGLES, University of Wisconsin Carbone Cancer Center, Madison, Wisconsin.

## Abstract

**Introduction:**

Cancer incidence and mortality rates for American Indians in the Northern Plains region of the United States are among the highest in the nation. Reliable cancer surveillance data are essential to help reduce this burden; however, racial data in state cancer registries are often misclassified, and cases are often underreported.

**Methods:**

We used a community-based participatory research approach to conduct a retrospective ascertainment of cancer cases in clinic medical records over a 9-year period (1995–2003) and compared the results with the state cancer registry to evaluate missing or racially misclassified cases. Six tribal and/or urban Indian clinics participated in the study. The project team consisted of participating clinics, a state cancer registry, a comprehensive cancer center, an American Indian/Alaska Native Leadership Initiative on Cancer, and a set of diverse organizational partners. Clinic personnel were trained by project staff to accurately identify cancer cases in clinic records. These records were then matched with the state cancer registry to assess misclassification and underreporting.

**Results:**

Forty American Indian cases were identified that were either missing or misclassified in the state registry. Adding these cases to the registry increased the number of American Indian cases by 21.3% during the study period (*P* = .05).

**Conclusions:**

Our results indicate that direct reporting of cancer cases by tribal and urban Indian health clinics to a state cancer registry improved the quality of the data available for cancer surveillance. Higher-quality data can advance the efforts of cancer prevention and control stakeholders to address disparities in Native communities.

## Introduction

American Indians and Alaska Natives (AI/ANs) often bear a greater cancer burden than other population groups (1–6). Cancer incidence and mortality rates for American Indians in the Northern Plains region of the United States, which includes Wisconsin, are among the highest in the nation (4,7,8). Evidence also shows that many AI/AN cancer cases may be misclassified in medical records, underreported to cancer registries, or both (9–15). Studies suggest the discrepancies may happen during the hospital intake process when a patient’s race and ethnicity are being recorded, because cancer patients may not accurately report their race/ethnicity or clinic staff erroneously document a patient’s race/ethnicity on the basis of cues such as age, marital status, or language use (13,16). Regardless of how these inaccuracies in the data arise, such discrepancies can greatly affect surveillance efforts and present additional barriers to addressing cancer disparities, particularly in small populations.

The objective of this community-based participatory research project was to assess AI/AN cancer case underreporting and misclassification in Wisconsin and to quantify the extent of any inaccuracies. This article reports on the second phase of the Improving American Indian Cancer Surveillance and Data Reporting in Wisconsin study, which was supported through the Great Lakes Native American Research Center for Health (NARCH) and led by Spirit of EAGLES: AI/AN Leadership Initiative on Cancer, a national effort to decrease cancer disparities funded by the National Cancer Institute. The initial phase of this study compared state registry data with national data from the Indian Health Service and found a significant number of misclassifications (17). The partners in this project saw potential for additional improvements in data accuracy by fostering reciprocal reporting relationships between local tribal health facilities and urban Indian clinics and the state cancer registry. In the present phase of the study, cancer case records from clinics serving AI/AN populations were compared with Wisconsin Cancer Reporting System case data. We sought to answer 2 questions: 1) Does the state cancer registry capture all cancer cases identified in tribal/urban clinic records; and, if so, 2) Is the race of AI/AN cancer patients correctly identified in the case records?

## Methods

Recognizing the importance of community participation (18–20), we conducted this research project collaboratively among multiple community and partner organizations. In cooperation with tribal and urban Indian clinic partners, the Great Lakes Inter-Tribal Epidemiology Center, the Wisconsin Cancer Reporting System, and the University of Wisconsin Carbone Cancer Center jointly assessed cancer case misclassification of AI/AN populations in Wisconsin. These partnerships were convened and facilitated by Spirit of EAGLES; the Carbone Cancer Center has had a Spirit of EAGLES subcontract since 2000. The overall objective was to develop long-term methods to improve the quality, completeness, and use of cancer data by American Indian tribes in Wisconsin.

Ten of 13 Wisconsin tribal and urban Indian clinics participated in the full study, and 6 clinics were involved in the case-matching endeavor described in this article. Clinics that participated in the matching phase of the study did not differ in any obvious ways from those who declined. Both participating and nonparticipating clinics were diverse in terms of tribal nation affiliations, community size, socioeconomic status, rurality, and geography. The most common reason given for not participating was a perceived lack of time on the part of the clinic staff.

In the initial phase, project staff from Spirit of EAGLES provided training to tribal and urban Indian clinic personnel at each of the participating clinic sites. The personnel were taught cancer case abstraction in accordance with the state cancer registry data collection standards. Training included instructing clinic personnel on how to use medical records to verify a diagnostic confirmation (eg, “microscopic confirmation”), identify the stages of the cancer, and identify treatment therapies, if applicable. The clinic staff was given additional training in the use of International Statistical Classification of Diseases and Related Health Problems cancer codes (ie, ICD-9 codes 140.0–208.9). After completing training, personnel searched clinic electronic medical manager systems for relevant ICD-9 codes, performed a manual chart audit of each case identified in the electronic medical manager systems, and collected abstracted data on project-specific forms. The forms were based on the standard state registry neoplasm record form that all Wisconsin hospitals and clinics were using at that time to report cancer cases. In keeping with the participatory nature of the project, staff from each clinic reviewed these forms for usability and added questions of local interest as desired.

Following the abstraction process, clinics forwarded the completed form and case information directly to the Wisconsin Cancer Reporting System. The data were then linked to the registry case records using unique patient identifiers. To accomplish this linkage, registry staff keyed the clinic data into electronic abstracting software that standardized and formatted it to allow for matching to the registry’s database. Next, registry staff searched for various combinations of identifiers (ie, name, date of birth, Social Security number, and home address) to obtain the best possible match between the clinics’ case information and the state registry. Exact data and “fuzzy” data (eg, year of birth, middle initial) were used to account for common keying errors in both incoming and registry data (eg, reversed month/day and reversed first/middle name). If there was a discrepancy in classification of race/ethnicity between the 2 data sources, the clinic data was typically given preference.

Clinic sites differed on which year services were first offered. Consequently, clinics had different time frames in which cancer cases were queried. For this study, data on cases diagnosed from 1995 through 2003 were chosen for analysis. This was the common interval of available cases across most clinic sites and was also the common interval available in the state registry at the time. Because of varying factors including data cleaning and cross-checking processes, case data in the state registry are typically 3 or more years behind at any given time. In late 2008 when this matching process was conducted, the registry had complete data available until 2003.

The 6 participating clinics submitted 419 cancer cases for linkage with the Wisconsin Cancer Reporting System. Fifty-six (13.4%) of the 419 cases had an unknown date of diagnosis, and 97 (23.2%) had a date of diagnosis either before 1995 or after 2003 and were excluded from analysis. The linkage with the state registry was conducted using the remaining 266 cases (Figure). Data from 1 clinic were excluded because all observations fell outside the time window or lacked dates of diagnosis. 

**Figure:**
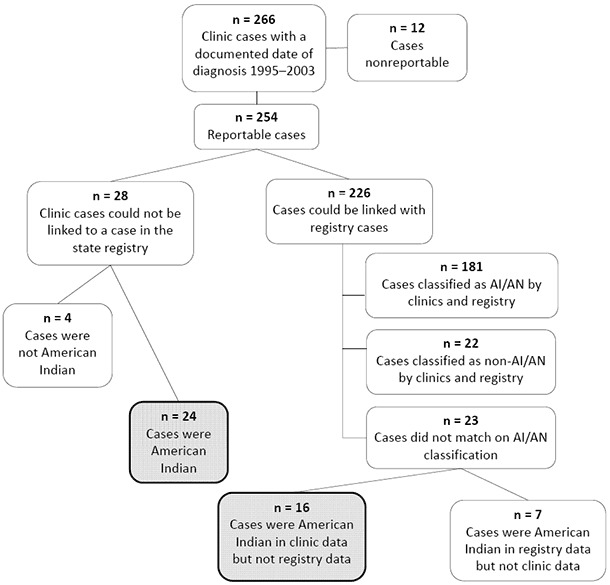
Breakdown of the cancer case matching process between participating Wisconsin tribal clinics and the Wisconsin Cancer Reporting System. Abbreviation: AI/AN, American Indian/Alaska Native.

Data analyses were completed using SAS 9.2 (SAS Institute, Inc, Cary, North Carolina). We used a Pearson χ^2^ statistic, because it is designed to test whether the difference between an established distribution (ie, the prematched AI/AN cancer cases in the state registry) and a new sample distribution (ie, the postmatched numbers of AI/AN cancer cases) is due to chance alone. This test was used to assess whether the change in the number of AI/AN cases in the state registry after the match was significant. Significance was set at *P* ≤ .05.

## Results

Overall, 266 cases with a documented date of diagnosis were found in the records of the clinics (Figure). Of these, almost all (n = 254, 95.5%) were reportable by Wisconsin law, although a few were nonreportable (ie, skin cancers, hemangiomas, and intracranial lipomas [n = 12, 4.5%]) and were excluded from further analyses. Among the reportable cases, 2 categories of case status were created: 1) clinic cases matching a registry report (n = 226, 85.0%) and 2) reportable cases not found in the registry database (n = 28, 10.5%). 

Of the 254 reportable clinic cases, 40 American Indian cases (15.7%) were either misclassified in the Wisconsin Cancer Reporting System (n = 16) or were missing from the registry altogether (n = 24). These 40 cases are indicated in the Figure by the 2 shaded cells. With these cases added to the state registry, the number of AI/AN cancer cases in the registry’s database increased from 188 to 228 for the study period, a significant change of 21.3% (*P* = .05).

## Discussion

Our findings show that, before matching, the cancer cases reported to the state cancer registry provided an incomplete characterization of Wisconsin’s AI/AN cancer burden. Although the actual number of newly identified cases was not large — 40 cases from 5 clinics from 1995 through 2003 — it was relevant to this population, resulting in a significant increase in the number of AI/AN cancer cases in the state registry for this period. A small number of newly identified cases affects ongoing surveillance and evaluation. As our study illustrates, direct reporting by tribal/urban clinics can improve the quality of cancer data and may have value for tribes and registries in other states that wish to improve surveillance of AI/AN cancer or work to reduce cancer disparities.

Nationally, tribal and urban Indian centers have become more engaged in cancer surveillance activities through Spirit of EAGLES, the Indian Health Service Division of Epidemiology and Disease Prevention, Centers for Disease Control and Prevention (CDC)–supported Tribal Comprehensive Cancer Control programs, and through other regional efforts, such as those of the Northwest Portland Area Indian Health Board and the New Mexico Tumor Registry. Many cancer control–focused educational and research efforts are ongoing nationally. For example, in Wisconsin, Spirit of EAGLES has convened a coalition of multiple partners including various American Indian tribes and organizations, the Wisconsin Well Woman Program, the Wisconsin Comprehensive Cancer Control Program, and the American Cancer Society. This coalition sponsors an annual statewide American Indian cancer conference for tribal members and is now in its tenth year. Additionally, the Menominee Nation partnered with University of Wisconsin School of Medicine and Public Health in a first-of-its-kind, tribally driven clinical trial on smoking cessation (21). Participants in these and other efforts across the country have repeatedly recognized the need for ongoing access to AI/AN cancer data, particularly the need for high-quality data at the local level. This study suggests one potential route — that is, direct reporting by tribal/urban clinics to the state cancer surveillance system — to achieving the goal of high-quality local cancer data.

In states without tribal clinics, assessing underreporting may be difficult but remains an important objective. In these states, the first step to assessing potential disparities in reporting is to compare the proportional distribution of the states’ AI/AN populations with national Indian Health Service estimates of cancer burden for that state or region. If it seems reasonable to suspect discrepancies (eg, if AI/AN populations appear to have rates well above or below what would be expected), partnering with state-recognized tribes or local organizations that serve AI/AN populations is a potential next step. Organizations serving these communities can assist in creating strategies to find discrepancies in state data. For example, surnames common to the population of interest could be matched with state registry data to assess potential inconsistencies. Research on Asian American populations in California indicates that such surname list matching is a viable solution to racial/ethnic misclassification when other options are unavailable (22).

Although local cancer data are highly valued, development of the necessary infrastructure is challenging. This can be particularly true for smaller tribal and urban clinics that may lack personnel with the time or training to abstract cancer cases, have few external resources, and have competing priorities. However, recognizing the reciprocal benefits of improved AI/AN cancer data, local clinics and state cancer registries might partner to achieve this goal. Training and ongoing technical support from registry staff, as well as an efficient means for tribal/urban clinics to report their cases, could help mitigate local clinic limitations. In Wisconsin, electronic reporting systems are already in use at the Wisconsin Cancer Reporting System, opening the potential for integration with clinics across the state. Additionally, annual reports returned to the clinics by the registry could provide information to help the clinics monitor their population’s cancer burden, focus interventions, and build a local cancer registry, while simultaneously demonstrating the value of local reporting. For example, a new project led by the Medical College of Wisconsin is piloting such a reciprocal cancer surveillance relationship between the Wisconsin Cancer Reporting System and the Red Cliff Tribal health facility. Efforts like these are critical to ensure the local high-quality cancer data needed to conduct effective surveillance and reduce cancer health disparities.

This study has both strengths and potential limitations. First, the small number of total cases impeded analysis of additional variables that may have increased our understanding of factors associated with cases being missed or misclassified. For example, patient sociodemographic characteristics and the type of cancer and treatment location may be related to the likelihood that these cases were properly documented, reported, or both. Our small sample size prohibited such exploration. Moreover, it remains unknown why some cases were missing from the registry given mandatory reporting by treating physicians in the state of Wisconsin. However, because tribal and urban Indian clinics are less likely than larger medical settings to treat cancers that they have diagnosed, it may be that these cases were not legally required to be reported. Additionally, the closest facility for a cancer patient to seek treatment may have been in a bordering state, so these cases may have been missed because the treating physician was not governed by Wisconsin law. The study was also limited by the use of data from 1995 through 2003 rather than more recent data. Classification of race may have improved since that time; if so, we would not be able to see the effect of this in our data. Regardless, improving historical data is still useful to strengthen ongoing surveillance and evaluation efforts. An additional limitation of this study was that the generalizability of the findings to all of Wisconsin or to other states may be limited, because not all tribal/urban clinics in the state participated in the study. However, even with limited participation, the matching of clinic data with the Wisconsin Cancer Reporting System still led to a significant improvement in the state-level data.

Our study had strengths as well. The collaborative and participatory process involving multiple organizations allowed for a unique assessment. Moreover, this project demonstrated the feasibility of working with multiple stakeholders to accomplish mutually beneficial ends. By involving local clinics in the project and linking them to resources and training, this project built additional capacity in these communities, which may help them in accomplishing their future data-related goals. Finally, this project not only answered a relevant research question but also accomplished the practical task of improving the state-level cancer registry data, which will benefit future cancer research and surveillance in Wisconsin.

The reporting of new cancer cases to the state cancer registry by American Indian clinics improved the quality of Wisconsin AI/AN cancer data above and beyond standard reporting practices and the previous linkage with national Indian Health Service records. Ongoing reporting should continue to improve data completeness and accuracy. Both the Wisconsin Cancer Reporting System and local tribal and urban Indian clinics have a stake in quality cancer data. Better cancer data will provide a superior foundation for focused cancer prevention and control efforts, informed advocacy for appropriate funding from state and federal sources to address AI/AN cancer disparities, and more accurate data for identifying research questions and conducting epidemiological studies. This study also provides a potential model for other state cancer registries to work with local tribal/urban clinics to increase the accuracy of their cancer data.
